# Evolutionary Mahalanobis Distance-Based Oversampling for Multi-Class Imbalanced Data Classification

**DOI:** 10.3390/s21196616

**Published:** 2021-10-04

**Authors:** Leehter Yao, Tung-Bin Lin

**Affiliations:** Department of Electrical Engineering, National Taipei University of Technology, Taipei 10618, Taiwan; dark5027@gmail.com

**Keywords:** oversampling, mahalanobis distance, MOPSO, classification, minority class, ellipsoid

## Abstract

The number of sensing data are often imbalanced across data classes, for which oversampling on the minority class is an effective remedy. In this paper, an effective oversampling method called evolutionary Mahalanobis distance oversampling (EMDO) is proposed for multi-class imbalanced data classification. EMDO utilizes a set of ellipsoids to approximate the decision regions of the minority class. Furthermore, multi-objective particle swarm optimization (MOPSO) is integrated with the Gustafson–Kessel algorithm in EMDO to learn the size, center, and orientation of every ellipsoid. Synthetic minority samples are generated based on Mahalanobis distance within every ellipsoid. The number of synthetic minority samples generated by EMDO in every ellipsoid is determined based on the density of minority samples in every ellipsoid. The results of computer simulations conducted herein indicate that EMDO outperforms most of the widely used oversampling schemes.

## 1. Introduction

With advancements in sensor technology and the Internet of things (IoT), vast quantities of sensing data have been collected and analyzed for different applications. Cost-effective sensors are widely used in our everyday lives to collect various types of data for further online or offline analyses and applications. The classification of real-world sensing data is a highly important research topic in the field of data mining and machine learning. However, the data sets collected using sensors or other sensing techniques usually have a skewed class distribution because the number of data points vary greatly between classes. Such data are called imbalanced data. The data utilized in applications, such as anomaly detection in high-speed trains [1,2,3], fault diagnosis of motors [4,5,6], fault detection and diagnosis in manufacturing processes [7,8,9], and medical diagnosis [10,11,12], are usually imbalanced. In imbalanced data sets, at least one class of data has significantly more data points compared with other classes. Learning on imbalanced data results in poor performance, and this problem has thus attracted considerable research attention in recent years. It is mainly because the performance of many conventional learning algorithms is degraded on the skewed class distribution of imbalanced data sets [13].

Balanced class distribution [14] or equal weighting of classification errors for every class [13] is generally assumed in most conventional machine learning algorithms. For instance, 95% and 5% of imbalanced data sets can comprise majority and minority class samples, respectively. With the equal weighting of the classification errors, traditional classification approaches tend to overlook several or most of the minority class samples in the attempt to minimize the overall classification error. Consequently, although the overall classification error rate is low, the classification error rate for the minority class is high. Minority class samples are important in classification in certain applications, such as medical diagnosis, anomaly detection, and fault detection and diagnosis. Majority class samples usually represent normal conditions, and minority class samples represent abnormal conditions, which can be key in such applications. The learning approaches for imbalanced data are designed to increase learning accuracy with respect to minority classes without trading off learning accuracy with respect to majority classes.

The learning approaches for imbalanced data can generally be categorized into three types: cost-sensitive learning, data-level learning, and ensemble learning, which are comprehensively reviewed in [15,16]. Cost-sensitive learning assigns higher misclassification costs to minority class samples than to majority class samples. Studies have proposed various learning approaches that adjust the misclassification cost using kernel functions; these approaches involve the radial basis function [17], matrix-based kernel regression [18], support vector machine [19,20], and deep learning [21,22].

Data-level learning approaches essentially rebalance the skewed data distribution of different classes by removing several majority class samples or by adding new minority class samples, and they can generally be divided into undersampling and oversampling learning approaches. The main advantage of data-level learning approaches is that they are independent of classifiers. They can be considered a type of data preprocessing approach. Therefore, data-level learning approaches can be easily integrated with other imbalanced learning approaches. Undersampling involves removing the majority class samples to ensure that the learning results are not overly biased toward the majority class [23,24,25,26]. Undersampling reduces both the number of samples in and the computational cost of machine learning. However, it tends to reduce the model’s capability to recognize majority classes. By contrast, oversampling involves increasing the number of minority class samples by resampling them or by generating synthetic samples. However, resampling by simply replicating the minority class samples does not improve learning of the decision region of minority classes. The synthetic minority oversampling technique (SMOTE) proposed in [27] selects several samples from a minority class. Searching in the vicinity of the selected samples, it identifies other samples of the minority class to generate new synthetic samples linearly between the two points. SMOTE is the most widely used oversampling technique because of its computational inexpensiveness. However, SMOTE is prone to overgeneralization because it synthesizes new samples through the random selection of minority class samples. Various adaptive sampling methods based on SMOTE have been proposed to overcome its limitation. The adaptive synthetic sampling approach for imbalanced learning algorithm (ADASYN) [28], SMOTEBoost [29], and Borderline SMOTE [30] are effective modified versions of SMOTE. In contrast to SMOTE, other algorithms, such as those in [31,32,33], have been proposed; these algorithms generate synthetic samples by learning the structure underlying the minority samples.

Ensemble learning for incomplete data integrates traditional machine learning approaches, such as boosting [34], bagging [35], and stacking [36], with other cost-sensitive or data resampling imbalanced learning approaches. In [37], SMOTE was integrated with Adaboost [38] to increase the number of minority samples and to assign higher weights to misclassified minority samples. A similar integration of Adaboost with a novel synthetic sampling method was proposed in [39]. The performance of boosting, bagging, and other hybrid techniques applied to imbalanced data has been compared in [40] and [41].

In the methods proposed in [31,32,33], synthetic samples are generated based not on individual minority samples as proposed in SMOTE [30] but on the underlying structure of the minority samples. Recently, a similar oversampling approach called Mahalanobis distance-based oversampling (MDO) was proposed in [42]. MDO generates synthetic samples based on the structure of the principal component space of minority samples. The synthetic samples generated by MDO have the same Mahalanobis distance as that of the considered minority sample. Because the class mean of the synthetic samples generated by MDO is the same as that of the minority class samples, the covariance structure of the minority class samples is preserved. In [43], a scheme called adaptive Mahalanobis distance oversampling (AMDO) was proposed. AMDO integrates generalized singular value decomposition with MDO to solve the oversampling problem encountered in mixed-type imbalanced data sets. Either MDO or AMDO can be utilized as a direct learning approach for solving problems with multi-class imbalanced problems.

The oversampling results obtained from MDO or AMDO are equivalent to those obtained by placing minority class samples and generated synthetic samples into the principal component space. The minority class samples and the synthetic samples can be considered to be included in an ellipsoid centered at the class mean. The orientation of the ellipsoid depends on the covariance structure of the minority class samples. The synthetic samples do not change the covariance structure of the minority class because all the synthetic samples are generated within the ellipsoid. However, both MDO and AMDO use only one ellipsoid to include the minority class samples and synthetic samples. If the decision regions of the minority class are separated, the decision region approximated using only one ellipsoid may overlap with the decision regions of other classes. This is especially true for imbalanced multi-class data. Samples from different classes may be included in a single ellipsoid structure depending on the target minority class samples. The synthetic samples generated by MDO or AMDO are randomly assigned in the single ellipsoid only if they have the same Mahalanobis distance as that of the associated minority sample. When synthetic samples are generated within a single ellipsoid, the generated synthetic samples tend to be placed in the cluster of samples belonging to other classes. This reduces the effectiveness of oversampling. Moreover, certain decision regions (e.g., those that are ring- or belt-shaped) are difficult to approximate with only one ellipsoid.

A novel approach called evolutionary Mahalanobis distance oversampling (EMDO) is proposed in this paper to overcome the limitations of MDO and AMDO. EMDO utilizes multiple ellipsoids to learn the distribution and orientation of minority class samples in parallel. Gustafson and Kessel proposed a clustering algorithm called the Gustafson–Kessel algorithm (GKA) [44], which is similar to the widely used fuzzy *c*-means [45] clustering approach with Mahalanobis norms. The advantage of the GKA over fuzzy c-means is that it utilizes the Mahalanobis norm instead of the Euclidean norm to learn the underlying sample distribution. However, the GKA assumes a fixed volume before learning the center and orientation of every ellipsoid. The GKA is an effective clustering approach for learning the centers and orientations of data clusters, but it is unsuitable for learning the decision regions of data due to its assumption of a fixed ellipsoid size. The GKA was modified in [46,47] to adaptively learn ellipsoid sizes for pattern recognition problems by using the genetic algorithm with a single objective function. In the proposed EMDO, the GKA is integrated with multi-objective particle swarm optimization (MOPSO) [48,49] to ensure that the centers, orientations, and sizes of multiple ellipsoids, along with the overall misclassification error, are learned in parallel. The misclassification error is defined as the total number of misclassified samples included in a union of multiple ellipsoids. Therefore, EMDO can learn a set of ellipsoids to approximate connected or disconnected complex decision regions with reasonable accuracy. Because multiple ellipsoids are learned in parallel in EMDO, an effective approach is designed to adaptively determine the number of synthetic samples to be generated in every ellipsoid. Similar ideas that design suitable algorithms to search for model parameters for specific applications are shown in [50,51,52].

The technical novelty and main contribution of this paper are summarized as follows.

1) An effective novel oversampling approach called EMDO is proposed for multi-class imbalanced data problems. Different from the MDO and AMDO approaches that use only one ellipsoid, EMDO learns multiple ellipsoids in parallel to approximate the decision region of the target minority class samples.
2) MOPSO is utilized along with GKA in EMDO to optimize the parameters, including the centers, orientations, and sizes of multiple ellipsoids approximating the target class of decision regions with reasonable accuracy.
3) Synthetic minority samples are generated based on the Mahalanobis distance within every ellipsoid learned by EMDO. A novel adaptive approach is proposed to determine the number of synthetic minority samples to be generated based on the density of minority samples in every ellipsoid.
4) EMDO was evaluated and found to perform better than other widely used oversampling schemes.

The remainder of this paper is organized as follows. Section 2 presents the problem formulation of oversampling for imbalanced data. The GKA is introduced in this section, and it shows that the GKA is suitable to solve the problem formulated herein. Section 3 introduces the proposed multi-objective optimization scheme designed in the EMDO, which uses MOPSO. Section 4 details the method for calculating the number of ellipsoids required to approximate the decision regions of every class. Section 5 describes performance evaluation of EMDO against other widely used oversampling schemes. Finally, Section 6 concludes the study.

## 2. Problem Statement and GKA

Given a data set $S=\{\left.\left(x_{i},y_{i}\right)\right|x_{i}\in R^{d},\;y_{i}\in \{1\ldots p\},i=1\ldots N\}$, every *i*th sample $x_{i}\in S$ belongs to some class $y_{i}$ among *p* classes. Let $S_{j}\subset S$ be the set containing the samples belonging to class *j*, *j* = 1…*p*. Denote $N_{j}\equiv \left|S_{j}\right|$ as the number of samples in $S_{j}$, where $N_{\min}=\underset{j=1\ldots p}{\min}(N_{j})$, $N_{\max}=\underset{j=1\ldots p}{\max}(N_{j})$. The data set ***S*** is imbalanced if $\left(N_{j}/N_{\max}\right)$ is less than a preset imbalance ratio, IR. The value of IR is determined based on the size of ***S*** and on the characteristics of the classification problem. Typically, IR ≥ 1.5. $S_{j}$ is called a minority set if $(\left|S_{j}\right|/N_{\max})<IR$, *j* = 1…*p*. An oversampling technique is applied in this study to overcome the skewed distribution of samples in ***S***. If $S_{j}$ is a minority set, the synthetic samples belonging to the same *j*th class are generated in $S_{j}$ to form an enlarged set ${\tilde{S}}_{j}$ such that $\left|{\tilde{S}}_{j}\right|=N_{\max}/IR$. Denote ${\tilde{N}}_{j}$ as the total number of extra synthetic samples generated to balance the minority set $S_{j}$,

(1) $${\tilde{N}}_{j}=(N_{\max}/IR-\left|S_{j}\right|).$$

Note that there can be more than one minority set in a multi-class problem. An oversampling technique is proposed herein to improve classification accuracy on an imbalanced data set. To generate an adequate number of synthetic samples and place them in the minority sets, the distribution of decision regions of every minority set in the *d*-dimensional feature space must be located. Multiple ellipsoids are utilized in this study to approximate the decision regions of minority sets. EMDO is proposed to learn these ellipsoids and generate synthetic samples in these ellipsoids for oversampling.

Assume that ellipsoids approximate the decision region of the *j*th class samples. Denote the center of every *n*th ellipsoid as $v_{n}^{j}\in R^{d}$. The distance between every *k*th sample $x_{k}$ and the ellipsoid center $v_{n}^{j}$ is defined in the Mahalanobis form as follows:

(2) $$\lambda_{nk}^{j}={\left({\left(x_{k}-v_{n}^{j}\right)}^{T}M_{n}^{j}(x_{k}-v_{n}^{j})\right)}^{1/2},$$

where $M_{n}^{j}\in R^{d\times d}$ is a norm-inducing matrix. The ellipsoid $\Phi_{n}^{j}$ is defined as follows by using the Mahalanobis distance defined in (2):

(3) $$\Phi_{n}^{j}(x_{k})={\left(x_{k}-v_{n}^{j}\right)}^{T}M_{n}^{j}(x_{k}-v_{n}^{j})=1.$$

The sample $x_{k}$ is inside or on the ellipsoid if $\Phi_{n}^{j}(x_{k})\leq 1$, but it is outside the ellipsoid if $\Phi_{n}^{j}(x_{k})>1$. Let the decision region of the *j*th class samples in the feature space be denoted as ${ℜ}^{j}$; ${ℜ}^{j}$ is approximated by the union of $\alpha^{j}$ ellipsoids, that is,

(4) $${ℜ}^{j}≅\underset{n=1\ldots \alpha^{j}}{\cup }\Phi_{n}^{j}.$$

The GKA is used for learning the $\alpha^{j}$ ellipsoids in parallel, given that the size of each ellipsoid is assigned. Denote the size of the ellipsoid $\Phi_{n}^{j}$ in (3) as $\xi_{n}^{j}$, *n* = 1…$\alpha^{j}$. The determinant of the norm-inducing matrix $M_{n}^{j}$ is inversely proportional to $\xi_{n}^{j}$. Therefore,

(5) $$\det(M_{n}^{j})=1/\xi_{n}^{j},\;n=1\ldots \alpha^{j}.$$

The GKA learns the norm-inducing matrices $M_{n}^{j}$ and the ellipsoid centers $v_{n}^{j}$ through iteratively calculating an auxiliary fuzzy partition matrix $U^{j}\in R^{\alpha^{j}\times N^{j}}$ by using all $N^{j}$ samples belonging to the *j*th class. The element $\mu_{nk}^{j}\in U^{j}$ represents the membership value of the *k*th sample $x_{k}$ associated with the *n*th ellipsoid $\Phi_{n}^{j}$. The membership values sum to 1 for every $x_{k}$, that is,

(6) $$\sum_{n=1}^{\alpha^{j}}\mu_{nk}^{j}=1,\;k=1\ldots N^{j}.$$

The GKA is a fast iterative learning algorithm that efficiently updates the membership values in the fuzzy partition matrix $U^{j}$ while learning both the norm-inducing matrix $M_{n}^{j}$ and the center $v_{n}^{j}$ of every *n*th ellipsoid. Note that the GKA learns all $\alpha^{j}$ ellipsoids in parallel. Denote the matrices containing the ellipsoid centers and the norm-inducing matrices as $V^{j}$ and $M^{j}$, respectively, that is, $V^{j}=[v_{1}^{j},v_{2}^{j}\ldots ,v_{\alpha^{j}}^{j}]$ and $M^{j}=[M_{1}^{j},M_{2}^{j},\ldots ,M_{\alpha^{j}}^{j}]$. All elements in the triple $\left(U^{j},V^{j},M^{j}\right)$ are learned by iteratively minimizing the distance in (2) weighted with the membership values in $U^{j}$ subject to the constraints in (5) and (6). Let $\omega_{n}^{j}$, *n* = 1…$\alpha^{j}$ and $\varpi_{k}^{j}$, *k* = 1…$N^{j}$ be the Lagrange multipliers of the constraints in (5) and (6), respectively. The triple $\left(U^{j},V^{j},M^{j}\right)$ is iteratively learned as follows:

(7) $$(U^{j},V^{j},M^{j})=\arg\min(\sum_{n=1}^{\alpha^{j}}\sum_{k=1}^{N^{j}}{\left(\mu_{nk}^{j}\right)}^{b}{\left(\lambda_{nk}^{j}\right)}^{2}+\sum_{n=1}^{\alpha^{j}}\omega_{n}^{j}(\det(M_{n}^{j})-1/\xi_{n}^{j})+\sum_{k=1}^{N^{j}}\varpi_{k}^{j}(\sum_{n=1}^{\alpha^{j}}\left(\mu_{nk}^{j}-1)\right),\;$$

where *b* is an adjusted weighting index. The optimization described in (7) is realized by differentiating (7) with respect to $\mu_{nk}^{j},\;v_{n}^{j},\;\omega_{n}^{j}$, and $\varpi_{k}^{j}$,and by equating the result to 0.

The parameters are obtained as follows:

(8) $$\mu_{nk}^{j}=\frac{1}{\sum_{i=1}^{\alpha^{j}}{\left(\lambda_{nk}^{j}/\lambda_{ik}^{j}\right)}^{2/(b-1)}},\;n=1\ldots \alpha^{j},\;k=1\ldots N^{j};$$

(9) $$v_{n}^{j}=\frac{\sum_{k=1}^{N^{j}}{\left(\mu_{nk}^{j}\right)}^{b}x_{k}}{\sum_{k=1}^{N^{j}}{\left(\mu_{nk}^{j}\right)}^{b}},\;n=1\ldots \alpha^{j};$$

(10) $$F_{n}^{j}=\frac{\sum_{k=1}^{N^{j}}{\left(\mu_{nk}^{j}\right)}^{b}(x_{k}-v_{n}^{j}){\left(x_{k}-v_{n}^{j}\right)}^{T}}{\sum_{k=1}^{N^{j}}{\left(\mu_{nk}^{j}\right)}^{b}},\;n=1\ldots \alpha^{j};$$

(11) $$\mathrm{and}M_{n}^{j}={\left(\zeta_{n}^{j}\det(M_{n}^{j})\right)}^{1/d}{\left(M_{n}^{j}\right)}^{-1}\;n=1\ldots \alpha^{j}.$$

The iteration in GKA is stopped when no significant improvement is made in the fuzzy partition matrix $U^{j}$. Let ${\left(U^{j}\right)}^{\left(m\right)}$ be the fuzzy partition matrix learned in the *m*th iteration, the norm of the difference between ${\left(U^{j}\right)}^{\left(m\right)}$ and ${\left(U^{j}\right)}^{\left(m+1\right)}$ can be defined as

(12) $$\delta^{j}=\left\|{\left(U^{j}\right)}^{\left(m+1\right)}-{\left(U^{j}\right)}^{\left(m\right)}\right\|\equiv \underset{n,k}{\max}\left|{\left(\mu_{nk}^{j}\right)}^{\left(m+1\right)}-{\left(\mu_{nk}^{j}\right)}^{\left(m\right)}\right|.$$

**Figure 1:**
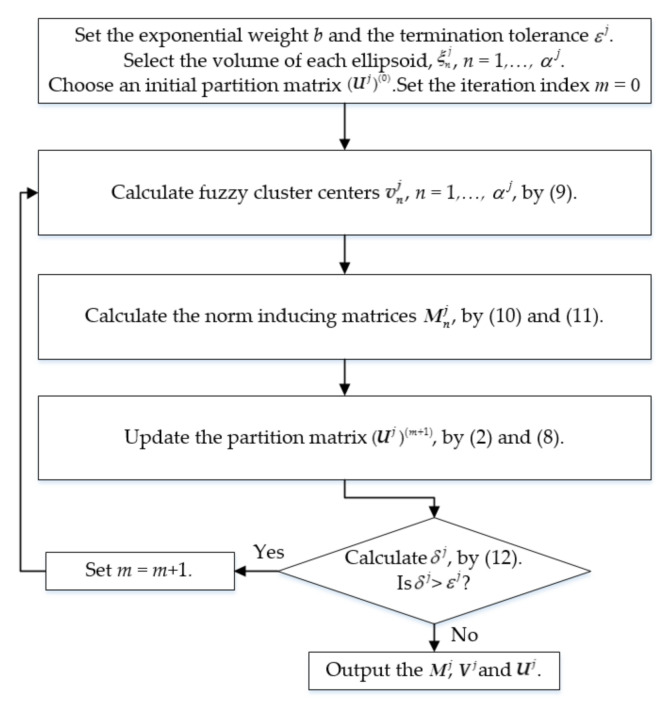
Flow chart of GKA.

The GKA iteratively learns $U^{j}$, $V^{j}$, and $M^{j}$ until $\delta^{j}<\varepsilon^{j}$, where $\varepsilon^{j}$ is a small constant. The flowchart of the GKA is illustrated in Figure 1.

## 3. Multi-Objective Optimization in EMDO

As depicted in Figure 1 and described in Section 2, the GKA optimizes the centers and norm-inducing matrices of multiple ellipsoids in parallel with a preset size of every ellipsoid. If the ellipsoid size is set inappropriately, the ellipsoids learned by the GKA cannot accurately include all minority class samples. According to (4), each *j*th-class decision region ${ℜ}^{j}$ is approximated by the union of $\alpha^{j}$ ellipsoids $\Phi_{n}^{j}$ of size $\xi_{n}^{j}$, *n* = 1…$\alpha^{j}$. Consider the sets $\Phi^{j}=\{\Phi_{1}^{j},\Phi_{2}^{j},\ldots ,\Phi_{\alpha^{j}}^{j}\}$ and $\Xi^{j}=\{\xi_{1}^{j},\xi_{2}^{j},\ldots ,\xi_{\alpha^{j}}^{j}\}$. The distance between the *k*th sample $x_{k}$ and ${ℜ}^{j}$, denoted $L(x_{k},{ℜ}^{j})$, can be defined as the minimum distance between $x_{k}$ and the center of each ellipsoid, as follows:

(13) $$L(x_{k},{ℜ}^{j})≅L(x_{k},\Phi^{j})=\underset{n=1\ldots \alpha^{j}}{\min}\lambda_{nk}^{j},$$

where $\lambda_{nk}^{j}$ is defined in (2). The sample $x_{k}$ is included in ${ℜ}^{j}$ if $L(x_{k},{ℜ}^{j})\leq 1$ and the corresponding class $y_{k}=j$. Denote a binary function H(⋅) as follows:

(14) $$H(ο)=\left\{\begin{array}{c} 1,\;\mathrm{if}\;\mathrm{the}\;\mathrm{logic}\;\mathrm{statement}\;ο\;\mathrm{is}\;\mathrm{true};\\ 0,\;\mathrm{if}\;\mathrm{the}\;\mathrm{logic}\;\mathrm{statement}\;ο\;\mathrm{is}\;\mathrm{false}.\; \end{array}\right.$$

The total number of *j*th-class samples included in the set $\Phi^{j}$ can be calculated as

(15) $$O_{included}^{j}(\Phi^{j})=\sum_{k=1}^{N}H(L(x_{k},\;\Phi^{j})\leq 1\;\mathrm{and}\;y_{k}=j).$$

It is possible that several samples that do not belong to the *j*th class are included in the set of ellipsoids $\Phi^{j}$. The total number of samples not belonging to the *j*th class but included in $\Phi^{j}$ can be calculated as

(16) $$O_{included}^{\backslash j}(\Phi^{j})=\sum_{k=1}^{N}H(L(x_{k},\;\Phi^{j})\leq 1\;\mathrm{and}\;y_{k}\neq j),$$

where $N_{included}^{\backslash j}(\cdot )\leq (N-N^{j})$.

Referring to (5), the total size of the ellipsoids contained in $\Phi^{j}$ can be calculated as

(17) $$F_{1}(\Xi^{j})=\sum_{n=1}^{\alpha^{j}}\xi_{n}^{j}.$$

The proposed EMDO not only minimizes the total ellipsoid sizes but also aims to simultaneously maximize the number of *j*th class samples included in the set of ellipsoids $\Phi^{j}$ and minimize the number of samples included in $\Phi^{j}$ but not belonging to *j*th class. The misclassification error can be defined as the summation of the number of *j*th class samples not included in $\Phi^{j}$, calculated as $\left(N^{j}-O_{included}^{j}({\left.\Phi^{j}\right|}_{\Xi^{j}}\right)$, and the number of samples that are included in $\Phi^{j}$ but that do not belong to the *j*th class is calculated as $O_{included}^{\backslash j}(\Phi^{j}{|}_{\Xi^{j}})$. Therefore, the misclassification error can be defined as

(18) $$F_{2}(\Phi^{j}{|}_{\Xi^{j}})=N^{j}-O_{included}^{j}(\Phi^{j}{|}_{\Xi^{j}})+O_{included}^{\backslash j}(\Phi^{j}{|}_{\Xi^{j}}).$$

The misclassification error can be utilized as an objective function to optimize the ellipsoid sizes. The set of ellipsoid sizes $\Xi^{j}=[\xi_{1}^{j},\xi_{2}^{j},\ldots ,\xi_{\alpha^{j}}^{j}]$ can be optimized using a multi-objective optimization scheme that minimizes both (17) and (18).

MOPSO is utilized to perform this multi-objective optimization by searching for the best set of ellipsoid sizes $\Xi^{j}$ by minimizing the objective functions $F_{1}(\cdot )$ and $F_{2}(\cdot )$. Assume that *G* particles are utilized in the MOPSO. Denote $\Xi^{j}(k,g)$ as the *g*th particle in the *k*th iteration and ${\bar{\Xi }}^{j}(g)$ as the non-dominated solution subject to the following multi-objective optimization. Note that the multi-objective optimization searches for the non-dominated solution of every particle.

(19) $${\bar{\Xi }}^{j}(g)=\underset{\Xi^{j}(k,g),\;\forall x^{k}\in S,\;g=1\ldots G}{Arg\min}(F_{1}(\Xi^{j}(k,g)),F_{2}(\Phi^{j}{|}_{\Xi^{j}(k,g)})).$$

$\Xi^{j}(k,g)$ is defined to be a non-dominated solution, as given by (19), if it is not dominated by any other particle, that is, if both $F_{1}(\Xi^{j}(k,g))\leq F_{1}(\Xi^{j}(k,i))$ and $F_{2}(\Phi^{j}{|}_{\Xi^{j}(k,g)})\leq F_{2}(\Phi^{j}{|}_{\Xi^{j}(k,i)})$, *i*=1…G, i≠g. Let

(20) $$a_{i}(\Xi^{j}(k,g))=H(F_{1}(\Xi^{j}(k,g))>F_{1}(\Xi^{j}(k,i))\;\mathrm{or}\;F_{2}(\Phi^{j}{|}_{\Xi^{j}(k,g)})>F_{2}(\Phi^{j}{|}_{\Xi^{j}(k,i)}))$$

(21) $$A(\Xi^{j}(k,g))=\sum_{i=1\ldots G,i\neq g}^{}a_{i}(\Xi^{j}(k,g)).$$

The non-dominated solution obtained using the *g*th particle, denoted as ${\bar{\Xi }}^{j}(g)$, is updated as $\Xi^{j}(k,g)$ if $A(\Xi^{j}(k,g))$=0. However, ${\bar{\Xi }}^{j}(g)$ remains unchanged if $A(\Xi^{j}(k,g))$>0.

(22) $${\bar{\Xi }}^{j}(g)=\left\{\begin{array}{c} \Xi^{j}(k,g),\;\mathrm{if}\;A(\Xi^{j}(k,g))=0;\\ {\bar{\Xi }}^{j}(g),\;\mathrm{otherwise}.\; \end{array}\right.$$

Only if $\Xi^{j}(k,g)$ is the non-dominated solution of (19) will it be included in the repository $S_{\mathrm{rep}}^{j}$, which is the set of all of non-dominated solutions of (19). The best solution achieved by the *g*th particle, denoted $\Xi_{p\_\mathrm{best}}^{j}(g)$, is updated using this non-dominated solution generated by the *g*th particle, that is, $\Xi_{p\_\mathrm{best}}^{j}(g)$ = ${\bar{\Xi }}^{j}(g)$. With reference to (22), if ${\bar{\Xi }}^{j}(g)$ is not generated in the current *k*th iteration, $\Xi_{p\_\mathrm{best}}^{j}(g)$ remains unchanged.

It is possible that several original non-dominated solutions stored in $S_{\mathrm{rep}}^{j}$ become dominated after a new non-dominated solution is included in $S_{\mathrm{rep}}^{j}$. A process for filtering out non-dominated solutions, similar to the one proposed in (20) and (21), is executed for all of the non-dominated solutions in $S_{\mathrm{rep}}^{j}$. Assume a total of $m_{rep}$ non-dominated solutions are in the repository, including the newly generated one. Each of the $m_{rep}$ non-dominated solutions is compared with all of the other solutions to evaluate whether they are being dominated. Denote ${\bar{\Xi }}_{m}^{j}$ as the *m*th non-dominated solution. Let

(23) $$a_{i}({\bar{\Xi }}_{m}^{j})=H(F_{1}({\bar{\Xi }}_{m}^{j})>F_{1}({\bar{\Xi }}_{i}^{j})\;\mathrm{or}\;F_{2}(\Phi^{j}{|}_{{\bar{\Xi }}_{m}^{j})})>F_{2}(\Phi^{j}{|}_{{\bar{\Xi }}_{i}^{j}}))$$

(24) $$A({\bar{\Xi }}_{m}^{j})=\sum_{i=1\ldots m_{rep},i\neq m}^{}a_{i}({\bar{\Xi }}_{m}^{j}).$$

${\bar{\Xi }}_{m}^{j}$ is no longer a non-dominated solution and is excluded from $S_{\mathrm{rep}}^{j}$ if $A({\bar{\Xi }}_{m}^{j})$ > 0.

An adaptive grid algorithm [53] is applied to $S_{\mathrm{rep}}^{j}$ after the filtering process is completed, as given in (23) and (24), to place all the non-dominated solutions into several grids. The global best particle $\Xi_{g\_\mathrm{best}}^{j}$ is randomly selected from among the grids in $S_{\mathrm{rep}}^{j}$ by using the roulette wheel selection scheme. After both $\Xi_{p\_\mathrm{best}}^{j}(g)$ and $\Xi_{g\_\mathrm{best}}^{j}$ are determined, each particle is updated as follows:

(25) $$\tau (k,g)=\tau (k-1,g)+c_{1}\gamma_{1}(\Xi_{p\_\mathrm{best}}^{j}(g)-\Xi^{j}(k,g))+c_{2}\gamma_{2}(\Xi_{g\_\mathrm{best}}^{j}-\Xi^{j}(k,g)),$$

(26) $$\Xi^{j}(k,g)=\Xi^{j}(k-1,g)+\tau (k,g),\;g=1\ldots G,$$

where $c_{1}$ and $c_{2}$ are preset constants and $\gamma_{1},\;\gamma_{2}\in [0,1]$ are randomly generated real numbers. If no new non-dominated solution can be successfully included in $S_{\mathrm{rep}}^{j}$ after the filtering process for certain preset $K_{thr}$ iterations, MOPSO is saturated, and the iterative learning of particles given by (19)–(26) is stopped.

The optimal solution of the multi-objective optimization problem in (19) is searched from $S_{\mathrm{rep}}^{j}$ after the iterative learning process is stopped. Let ${\bar{\Xi }}_{m}^{j}=\{{\bar{\xi }}_{m1}^{j},{\bar{\xi }}_{m2}^{j},\ldots ,{\bar{\xi }}_{m\alpha^{j}}^{j}\}$ be the *m*th non-dominated solution in $S_{\mathrm{rep}}^{j}$. The average density of the ellipsoids associated with ${\bar{\Xi }}_{m}^{j}$ is defined as

(27) $$d(\Phi^{j}{|}_{{\bar{\Xi }}_{m}^{j}})=\frac{O_{included}^{j}(\Phi^{j}{|}_{{\bar{\Xi }}_{m}^{j}})}{\sum_{n=1}^{\alpha^{j}}{\bar{\xi }}_{mn}^{j}}.$$

The optimal solution ${\left(\Xi^{j}\right)}^{*}$ can be selected based on the average density of the non-dominated solutions because the ellipsoids associated with the optimal solution tend to have a small size but a large number of samples. However, the average density in (27) cannot be directly utilized as the optimization index for selecting the optimal solution. It is modified as the ratio of the total number of *j*th class samples included in the set $\Phi^{j}$ multiplied with the ratio of the total number of samples not belonging to the *j*th class but included in $\Phi^{j}$. Let the evaluation index for the *mth* non-dominated solution ${\bar{\Xi }}_{m}^{j}$ be $\sigma^{j}({\bar{\Xi }}_{m}^{j})$, which is defined based on (27) as follows:

(28) $$\sigma^{j}({\bar{\Xi }}_{m}^{j})=d(\Phi^{j}{|}_{{\bar{\Xi }}_{m}^{j}})\times \frac{O_{included}^{j}(\Phi^{j}{|}_{{\bar{\Xi }}_{m}^{j}})}{N^{j}}\times \frac{\left(N-N^{j}-O_{included}^{\backslash j}(\Phi^{j}{|}_{{\bar{\Xi }}_{m}^{j}})\right)}{N-N^{j}},$$

where $N^{\backslash j}(\Phi^{j}{|}_{{\bar{\Xi }}_{m}^{j}})$ denotes the number of samples not belonging to the *j*th class but included in $\Phi^{j}$. The optimal solution ${\left(\Xi^{j}\right)}^{*}$ can be defined as the set of ellipsoid sizes that maximize the index $\sigma^{j}(\cdot )$, that is,

(29) $${\left(\Xi^{j}\right)}^{*}=\underset{{\bar{\Xi }}_{m}^{j},\;m=1\ldots m_{rep}}{Arg\max}\sigma^{j}({\bar{\Xi }}_{m}^{j}).$$

**Figure 2:**
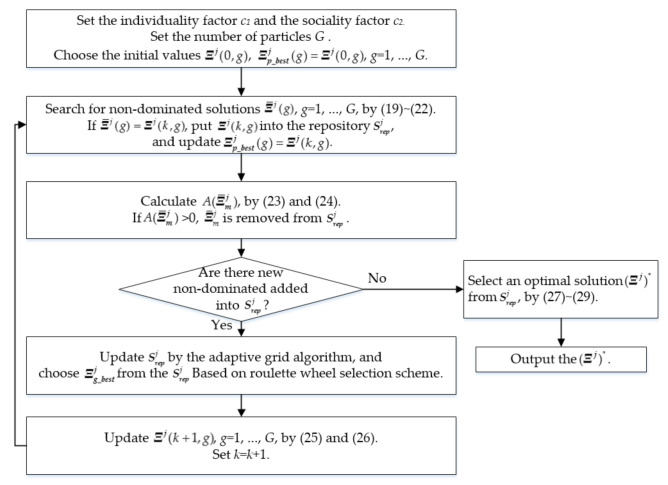
Flow chart of MOPSO.

After the optimal ellipsoid sizes are determined by MOPSO according to (19) and (29), the GKA is applied based on the optimal ellipsoid sizes ${\left(\Xi^{j}\right)}^{*}$ to calculate the other optimal ellipsoid parameters such as the norm-inducing matrix ${\left(M^{j}\right)}^{*}$ and the ellipsoid centers ${\left(V^{j}\right)}^{*}$. Note that the orientations of all the ellipsoids approximating the *j*th class decision are determined by the norm-inducing matrix ${\left(M^{j}\right)}^{*}$. The proposed MOPSO integrated with the GKA is illustrated in Figure 2.

## 4. Determining Number of Ellipsoids

**Figure 3:**
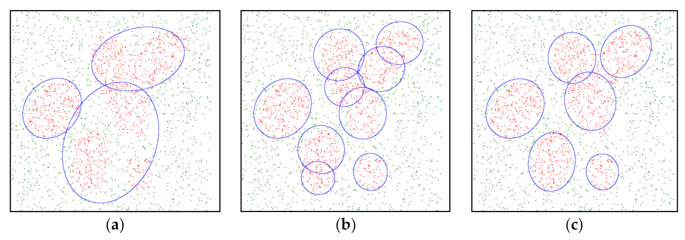
Learning results with different number of ellipsoids $\alpha^{j}$. (**a**) $\alpha^{j}$ = 3; (**b**) $\alpha^{j}$ = 9; (**c**) $\alpha^{j}$ = 6.

The ellipsoid parameters, such as size, centers, and orientation, are optimized using MOPSO integrated with the GKA, as described in Section 2 and Section 3. These ellipsoid parameters are calculated under the condition that the total number of ellipsoids $\alpha^{j}$ used to approximate the *j*th class decision region is assigned in advance *j* = 1…*p*. If $\alpha^{j}$ is too small, the *j*th class samples might be included in an insufficient number of ellipsoids, resulting in several ellipsoids having large sizes. It is possible that certain samples that do not belong to the *j*th class are included in these large ellipsoids. Moreover, samples other than those belonging to the *j*th class might be included because an insufficient number of ellipsoids is assigned to model the *j*th class samples. A binary-class dataset with 1000 samples in each class is illustrated in Figure 3. The samples in either class 1 or class 2 are randomly generated within the range [0, 3] on X and Y axis, respectively. The problem caused by a small $\alpha^{j}$ is illustrated in Figure 3a. Conversely, the *j*th class samples might be included in too many ellipsoids if $\alpha^{j}$ is too large. This results in a scenario where several ellipsoids overlap with each another, resulting in the ellipsoids being learned inefficiently, as illustrated in Figure 3b. If a suitable $\alpha^{j}$ is assigned, as illustrated in Figure 3c, the learning result leads to a set of ellipsoids with of the appropriate size, center, and orientation.

**Figure 4:**
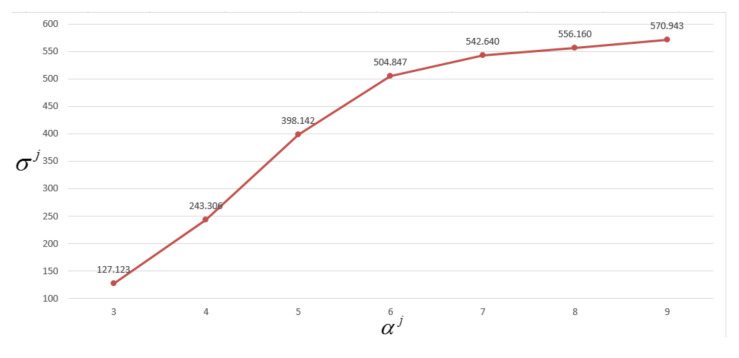
The typical curve of $\sigma^{j}({\left.{\left(\Xi^{j}\right)}^{*}\right|}_{\alpha^{j}=i})$ vs. $\alpha^{j}$.

To determine the suitable number of ellipsoids, the ellipsoid parameters are optimized using MOPSO integrated with the GKA by setting $\alpha^{j}$ from 1 to an appropriate number *q*. Denote ${\left.{\left(\Xi_{}^{j}\right)}^{*}\right|}_{\alpha_{}^{j}=i}$ as the optimal ellipsoid sizes calculated using MOPSO, according to (29), with $\alpha^{j}$ set to *i* ellipsoids, *i* = 1…*q*. The index $\sigma^{j}({\left.{\left(\Xi^{j}\right)}^{*}\right|}_{\alpha^{j}=i})$ in (28) is utilized to evaluate the effectiveness and efficiency for different numbers of ellipsoids $\alpha^{j}$. The suitable number of ellipsoids $\alpha^{j}$ can be determined at the value corresponding to the corner of the curve $\sigma^{j}({\left.{\left(\Xi^{j}\right)}^{*}\right|}_{\alpha^{j}=i})$ with respect to $\alpha^{j}$. Figure 4 shows a typical curve $\sigma^{j}({\left.{\left(\Xi^{j}\right)}^{*}\right|}_{\alpha^{j}=i})$ with respect to $\alpha^{j}$. According to this figure, $\alpha^{j}=6$ is a suitable choice because the curve corner appears at $\alpha^{j}=6$.

## 5. Generating Synthetic Samples

Any set of *j*th class samples with $(\left|S_{j}\right|/N_{\max})<IR$, *j* = 1…*p*, is considered a minority set. With reference to (1), ${\tilde{N}}_{j}$ synthetic samples are to be generated and added into the minority set. Recall that the minority set of the *j*th class samples is approximated by $\alpha^{j}$ ellipsoids. The ${\tilde{N}}_{j}$ synthetic samples must be proportionally added into each of the $\alpha^{j}$ ellipsoids based on the density $d_{n}^{j}$ of every *n*th ellipsoid, *n* = 1…$\alpha^{j}$. The number of *j*th class samples in the *n*th ellipsoid $\Phi_{n}^{j}$ is defined in a manner similar to (15) as

(30) $$O_{included}^{j}(\Phi_{n}^{j})=\sum_{k=1}^{N}H(\lambda_{nk}^{j}\leq 1\;\mathrm{and}\;y_{k}=j)$$

The number of samples included in the *n*th ellipsoid but not belonging to the *j*th class is

(31) $$O_{included}^{\backslash j}(\Phi_{n}^{j})=\sum_{k=1}^{N}H(\lambda_{nk}^{j}\leq 1\;\mathrm{and}\;y_{k}\neq j).$$

The density of ellipsoid $\Phi_{n}^{j}$ is defined as

(32) $$d(\Phi_{n}^{j})=\frac{O_{included}^{j}(\Phi_{n}^{j})}{\xi_{n}^{j}}.$$

The weight of the ellipsoid $\Phi_{n}^{j}$ for sharing the generated synthetic samples is defined as the reciprocal of the density $d(\Phi_{n}^{j})$ modified by the ratio of $O_{included}^{j}(\Phi_{n}^{j})$ to the total number samples in $\Phi_{n}^{j}$:

(33) $$\beta_{n}^{j}=\frac{1}{d(\Phi_{n}^{j})}\times \frac{O_{included}^{j}(\Phi_{n}^{j})}{O_{included}^{j}(\Phi_{n}^{j})+O_{included}^{\backslash j}(\Phi_{n}^{j})}.$$

Denote the number of synthetic samples added to $\Phi_{n}^{j}$ as ${\tilde{N}}_{n}^{j}$, which is determined based on the weight $\beta_{n}^{j}$ given in (33)

(34) $${\tilde{N}}_{n}^{j}={\tilde{N}}^{j}\times \frac{\beta_{n}^{j}}{\sum_{i=1}^{\alpha^{j}}\beta_{i}^{j}},\;n=1\ldots \alpha^{j}.$$

The scheme for generating synthetic samples for every ellipsoid $\Phi_{n}^{j}$ is designed to resolve the oversampling problem for the following two scenarios:

(A) $O_{included}^{j}(\Phi_{n}^{j})/(O_{included}^{j}(\Phi_{n}^{j})+O_{included}^{\backslash j}(\Phi_{n}^{j}))\geq 0.9$

In this case, more than 90% of the samples in $\Phi_{n}^{j}$ belong to the minority class. The samples other than those belonging to the *j*th class can be considered noise. The generated synthetic *j*th class samples do not affect the classification accuracy if they are randomly included in the ellipsoid $\Phi_{n}^{j}$. According to (2), $\Delta_{n}^{j}={\left(\lambda_{nk}^{j}\right)}^{2}={\left(x_{k}-v_{n}^{j}\right)}^{T}M_{n}^{j}(x_{k}-v_{n}^{j})$. Denote $z_{\mathrm{ni}}^{j}$ as the *i*th eigenvector of $M_{n}^{j}$ corresponding to the *i*th eigenvalue $\psi_{ni}^{j}$, *i* = 1…*d*. Let $Z_{n}^{j}=[z_{n1}^{j},\ldots ,z_{\mathrm{nd}}^{j}]$, $\Psi_{n}^{j}=diag(\psi_{n1}^{j},\ldots ,\psi_{nd}^{j})$. Because $M_{n}^{j}$ is a positive-definite matrix,

(35) $$\Delta_{n}^{j}={\left(\lambda_{nk}^{j}\right)}^{2}={\left(x_{k}-v_{n}^{j}\right)}^{T}M_{n}^{j}(x_{k}-v_{n}^{j}),\;n=1\ldots \alpha^{j}.$$

Let $p_{ni}^{j}={\left(z_{\mathrm{ni}}^{j}\right)}^{T}(x_{k}-v_{n}^{j})$, *i* =1…*d*; then, (35) can be rewritten as

(36) $$\Delta_{n}^{j}=\sum_{i=1}^{d}{\left(p_{ni}^{j}\right)}^{2}\psi_{ni}^{j},\;n=1\ldots \alpha^{j}.$$

Note that the sample $x_{k}$ is considered to be included in the ellipsoid if $\Delta_{n}^{j}\leq 1$. The ellipsoid has the center $v_{n}^{j}$, and all the eigenvectors $z_{\mathrm{ni}}^{j}$, *i* = 1…*d*, are orthogonal to one another. According to (36), every *i*th orthogonal eigenvector intersects the ellipsoid’s boundary sphere, where $\Delta_{n}^{j}=1$ at $1/\sqrt{\psi_{ni}^{j}}$ and $-1/\sqrt{\psi_{ni}^{j}}$.

The synthetic samples ${\hat{x}}_{k}^{j}$ are randomly generated in ellipsoid $\Phi_{n}^{j}$. The generated synthetic sample ${\hat{x}}_{k}^{j}$ is expressed as a linear combination of eigenvectors because all eigenvectors $z_{\mathrm{ni}}^{j}$ are orthogonal axes of the ellipsoid, that is,

(37) $${\hat{x}}_{k}^{j}=\sum_{i=1}^{d}z_{\mathrm{ni}}^{j}b_{ni},\;$$

where $b_{ni}$ is the projection of the vector $\left({\hat{x}}_{k}-v_{n}^{j}\right)$ onto the eigenvector $z_{\mathrm{ni}}^{j}$. To ensure random generation of the synthetic samples inside $\Phi_{n}^{j}$, $b_{ni}$ is set to be a random number within the range.

(38) $$-1/\sqrt{\psi_{ni}^{j}}\leq b_{ni}^{j}\leq 1/\sqrt{\psi_{ni}^{j}}$$

because each of the eigenvectors intersects the boundary sphere at $1/\sqrt{\psi_{ni}^{j}}$ and $-1/\sqrt{\psi_{ni}^{j}}$. However, the approach to randomly generate synthetic samples, as expressed in in (37) and (38), does not guarantee that the generated synthetic sample ${\hat{x}}_{k}^{j}$ is always in the ellipsoid $\Phi_{n}^{j}$. For every randomly generated ${\hat{x}}_{k}^{j}$, calculate the Mahalanobis distance according to (2) as

(39) $${\hat{\lambda }}_{nk}^{j}={\left({\left({\hat{x}}_{k}^{j}-v_{n}^{j}\right)}^{T}M_{n}^{j}({\hat{x}}_{k}^{j}-v_{n}^{j})\right)}^{1/2}.$$

The generated ${\hat{x}}_{k}^{j}$ is in $\Phi_{n}^{j}$ if ${\hat{\lambda }}_{nk}^{j}\leq 1$. No additional processing is required if ${\hat{x}}_{k}^{j}$ is in $\Phi_{n}^{j}$. The generated ${\hat{x}}_{k}^{j}$ is outside $\Phi_{n}^{j}$ if ${\hat{\lambda }}_{nk}^{j}>1$. Further processing is required if ${\hat{x}}_{k}^{j}$ is outside $\Phi_{n}^{j}$. The multiplication of ${\hat{x}}_{k}^{j}$ with a random number κ, where $0<\kappa \leq (1/{\hat{\lambda }}_{nk}^{j})$, leads to the multiplicative product $\kappa {\hat{x}}_{k}^{j}$ in $\Phi_{n}^{j}$. Denote the finally determined synthetic samples as ${\tilde{x}}_{k}^{j}$

(40) $${\tilde{x}}_{k}^{j}=\left\{\begin{array}{c} {\hat{x}}_{k}^{j},\;\mathrm{if}\;{\hat{\lambda }}_{nk}^{j}\leq 1;\\ \kappa_{1}{\hat{x}}_{k}^{j},\;\mathrm{if}\;{\hat{\lambda }}_{nk}^{j}>1; \end{array}\right.,$$

$\mathrm{where}\;\kappa_{1}\;\mathrm{is}\;a\;\mathrm{random}\;\mathrm{number}\;\mathrm{and}\;\kappa_{1}\in (0,1/{\hat{\lambda }}_{nk}^{j}].$(B) $O_{included}^{j}(\Phi_{n}^{j})/(O_{included}^{j}(\Phi_{n}^{j})+O_{included}^{\backslash j}(\Phi_{n}^{j}))<0.9$

In this case, more samples not belonging to the *j*th class are in $\Phi_{n}^{j}$. The random placement of synthetic samples in $\Phi_{n}^{j}$, as in the previous case, cannot effectively improve the classification accuracy. Borderline SMOTE [30] is modified to generate synthetic samples in this case. The samples located at the borderline between the clusters belonging and not belonging to the *j*th class must be first identified using Borderline SMOTE. For every *j*th class sample $x_{k}^{j}\in \Phi_{n}^{j}$, define the set $S_{k}^{j}$ containing all *m*-nearest neighbors. The *m*-nearest neighbors of $x_{k}^{j}$ are defined as the samples with the *m*-shortest Mahalanobis distances from $x_{k}^{j}$. Note that the Mahalanobis distance is calculated using the same norm-inducing matrix $M_{n}^{j}$ as in the case of $\Phi_{n}^{j}$, that is,

(41) $${\tilde{\lambda }}_{ki}^{j}={\left({\left(x_{k}^{j}-x_{i}\right)}^{T}M_{n}^{j}(x_{k}^{j}-x_{i})\right)}^{1/2},\;\forall x_{i}\in \Phi_{n}^{j},\;\mathrm{but}\;x_{i}\neq x_{k}^{j}.$$

The sample with all the *m*-nearest neighbors belonging to *j*th class is the sample not on the borderline. Conversely, the borderline sample contains at least one sample among the *m*-nearest neighbors not belonging to the *j*th class. Therefore, a sample is a borderline sample if at least one sample in the set of *m*-nearest neighbors $S_{k}^{j}$ does not belong to the *j*th class for every $x_{k}^{j}\in \Phi_{n}^{j}$.

After the borderline samples are identified, the synthetic samples are generated through random interpolation between the borderline sample $x_{k}^{j}$ and any other $x_{l}^{j}\in S_{k}^{j}$; that is, the synthetic sample is generated as follows:

(42) $${\tilde{x}}_{k}^{j}=x_{k}^{j}+\kappa_{2}(x_{k}^{j}-x_{l}^{j}),\;\forall x_{l}^{j}\in S_{k}^{j},$$

where $\kappa_{2}$ is a random number and $\kappa_{2}\in [0,1]$.

## 6. Simulation

The proposed EMDO was evaluated against other multi-class imbalanced data learning algorithms on different numerical data sets. The classifier C4.5 is usually utilized as the classifier to verify the oversampling results for various oversampling approaches. For instance, the oversampling approaches in [30,32,39], and [40,41,42,43] all used C4.5 as the classifier to verify the proposed oversampling schemes. This is mainly due to the fact that the classification results with C4.5 do not change as long as the parameter setting and datasets are fixed. No randomness exists in the classification results with C4.5 for the same parameter setting and dataset. Note that the number of ellipsoids utilized for the minority class is determined using the scheme proposed in Section 4 and is listed in the rightmost column of Table 1 and Table 6. The five nearest neighbors are considered for synthetic sample generation in case (B) of Section 5.

The simulations in this study were conducted using five-fold cross-validation with 10 independent runs. Every data set was tested using different oversampling schemes and compared with the proposed EMDO. The minority class with the minimum size was selected to validate the effectiveness and efficiency of the proposed EMDO.

Several evaluation metrics were designed to evaluate the effectiveness and efficiency of the proposed EMDO. The classification accuracy for the *j*th class is defined as follows:

(43) $$P_{j}=\frac{TP_{j}}{TP_{j}+FP_{j}},$$

where $TP_{j}$ is the number of true-positive classified samples, that is, the samples that are correctly classified as belonging to the *j*th class. $FP_{j}$ is the number of false-positive classified samples, that is, the samples that are incorrectly classified as belonging to the *j*th class. The metric $P_{avg}$ is defined as the average classification accuracy over all *p* classes, that is,

(44) $$P_{avg}=\frac{1}{p}\sum_{j=1}^{p}P_{j}.$$

The metric $P_{\min}$ refers to the classification accuracy defined in (43) for the minority class with the minimum size. To measure the capability of EMDO to separate any pair of classes, the area under curve (AUC) [54,55] is widely used in [56,57]. Denote $A_{m,n}$ as the AUC between class *m* and class *n*. The metric AUCm is defined as follows for measuring the capability of EMDO to separate the smallest minority class with the minimum size from the other classes.

(45) $$\mathrm{AUCm}=\frac{1}{p-1}\sum_{n\neq n’}\frac{A_{n,n’}+A_{n’,n}}{2}$$

where *n’* denotes the minority class with the minimum size. In addition to AUCm, the average of AUC over all pairs of classes for a multi-class problem, denoted as MAUC, is defined as

(46) $$\mathrm{MAUC}=\frac{2}{p(p-1)}\sum_{m<n}\frac{A_{m,n}+A_{n,m}}{2}.$$

In order to evaluate the imbalance condition of every data set, the maximum imbalance ratio IRmax is defined as follows:

(47) $$IR_{\max}=\frac{N_{\max}}{N_{\min}}$$

where $N_{\min}=\underset{j=1\ldots p}{\min}(N_{j})$, $N_{\max}=\underset{j=1\ldots p}{\max}(N_{j})$.

*Example 1:*

The data sets used in the simulation are the same as those used in [43] for comparing the performance of the EMDO against AMDO and other learning algorithms. The data sets used in [43] were mainly from data repositories such as the ones Knowledge Extraction based on Evolutionary Learning (KEEL) [58] and UCI (University of California, Irvine) Machine Learning Repository [59]. Table 1 describes these data sets. The performance comparison based on different indices are made in Table 2, Table 3, Table 4 and Table 5. Within Table 2, Table 3, Table 4 and Table 5, the algorithms such as SSMOTE refers to Static-SMOTE [60], GCS refers to RESCALE [61], ABNC refers to AdaBoost.NC [62], and OSMOTE refers to OVOSMOTE [63]. The MDO in [42], MDO+ and AMDO in [43] are also compared in Table 2, Table 3, Table 4 and Table 5 with the proposed EMDO. The Baseline algorithm is the classifier C4.5 without any oversampling technique.

To compare the performance of EMDO with those of the other schemes, the rank average of every scheme was calculated. All oversampling schemes were tested on each of the data sets listed in Table 1. The ranking of algorithm performance was based on each of the metrics. For instance, the algorithm with the best performance is ranked first, the algorithm with the second-to-best performance is ranked second, etc. The average rank of every algorithm is then calculated. The schemes with the same metric values share ranks. For instance, if two schemes are ranked second because they have the same metric values, the two schemes share the second and third ranks. These two schemes are thus ranked 2.5. The means and standard deviations of $P_{\min}$ and $P_{avg}$ are listed in Table 2 and Table 3, respectively, for every oversampling scheme, including the proposed EMDO, applied to different data sets. According to Table 2 and Table 3, EMDO outperforms all of the other schemes on every data set. EMDO has the lowest average rank. The results shown in both Table 2 and Table 3 imply that the oversampling performed using EMDO significantly improves the classification accuracy for the smallest minority class. Moreover, the synthetic samples generated for the minority class samples improve the overall average classification accuracy.

For all schemes, the mean and standard deviation are listed in Table 4 and the AUCm defined in (45) and MAUCm defined in (46) are also compared in Table 4 and Table 5, respectively. As indicated in Table 4, EMDO outperform the other schemes in separating the smallest minority class from the other classes for every listed data set. Moreover, according to Table 5, the capability of EMDO to separate all pairs of classes in the multi-class problem is superior to that of the other schemes.

*Example 2:*

The performance of EMDO is evaluated on the sensory data in this example. Two data sets, Statlog (Shuttle) from UCI and Mafalda [64] from Github are utilized in this example. The data set Statlog (Shuttle) is the set of the recorded sensory data from NASA’s space shuttle while the data set Mafalda is the set of the recorded sensory data from different brands of cars. The characteristics of these two data sets is shown in Table 6. Table 6 shows that both data sets are extremely imbalanced because the maximum imbalance ratios IR_max_ are as high as 5684.7 and 5.94, respectively. Four indices $P_{\min}$, $P_{avg}$, AUCm, and MAUC are calculated and compared in Table 7 for both data sets with classifier C4.5. The classification results are greatly improved with oversampling scheme EMDO compared with the results without EMDO. EMDO helps improve classification results for both highly imbalanced data sets according to the four evaluation indices listed in Table 7.

## 7. Conclusions

EMDO was demonstrated to outperform competing oversampling approaches in simulations. EMDO performed well because it approximates the decision region of the target minority class with reasonable accuracy by using a set of ellipsoids. In problems involving multi-class imbalanced data, EMDO performs exceptionally well if the decision region of the minority class is separated in the feature space. EMDO can learn the sizes, centers, and orientations of the ellipsoids that approximate the minority class decision region by using the underlying distribution of minority class samples. IoT is a key emerging technology, and imbalanced data will become an increasingly common problem as the number of IoT sensors increases. The proposed EMDO is suitable for solving such multi-class imbalanced data classification problems. One of the future works related to this study involves applying EMDO to address the problem of imbalanced data encountered in real-world IoT sensing data. Although EMDO is a data-level learning approach, it can easily be integrated with other cost-sensitive methods to increase the effectiveness and efficiency of learning. Further studies on variants of integration can be another direction for future research.

## Figures and Tables

**Table 1:** Characteristics of the data sets for simulations in Example 1.

Data Set	Size	Attributes	Classes	Class Distribution	IR_max_	No. of Ellipsoids
Balance	625	4	3	288/49/288	5.88	NA/4/NA
Hayes-Roth	132	4	3	51/51/30	1.7	NA/NA/3
New-Thyroid	215	5	3	150/35/30	5	NA/3/3
Page-Blocks	5472	10	5	4913/329/28/87/115	175.46	NA/9/4/6/9
Dermatology	358	34	6	111/60/71/48/48/20	5.55	NA/4/4/3/3/3
Breast-Tissue	106	9	6	21/15/18/16/14/22	1.57	NA/NA/NA/ NA/4/NA
User-Knowledge-Modelling (UKM)	403	5	5	50/102/129/122	2.58	4/NA/NA/NA
Vertebral-Column	310	6	3	60/150/100	2.5	6/NA/NA
Ecoli	327	7	5	143/77/52/35/20	7.15	NA/6/4/3/3

NA: not available.

**Table 2:** Comparison of $P_{\min}$ (%) for every over sampling scheme on different data sets.

Data Set	Baseline	SSMOTE	GCS	ABNC	OSMOTE	MDO	MDO+	AMDO	EMDO
Balance	0.00_0.00_	8.44_9.18_	6.44_9.83_	2.22_4.97_	12.44_13.41_	2.004.47	0.00_0.00_	10.22_0.50_	**20.41_10.95_**
Hayes-Roth	**100.00_0.00_**	**100.00_0.00_**	**100.00_0.00_**	**100.00_0.00_**	**100.00_0.00_**	**100.00_0.00_**	**100.00_0.00_**	**100.00_0.00_**	**100.00_0.00_**
New-Thyroid	83.33_11.79_	90.00_14.91_	93.33_9.13_	93.33_9.13_	90.00_9.13_	83.33_16.67_	96.67_7.45_	**100.00_0.00_**	**100.00_0.00_**
Page-Blocks	82.67_11.88_	78.67_6.91_	93.33_14.91_	72.67_24.99_	93.33_9.13_	75.33_16.26_	93.33_9.13_	**96.67_7.45_**	**96.67_5.33_**
Dermatology	95.00_11.18_	95.00_11.18_	90.00_13.69_	**100.00_0.00_**	90.00_13.69_	95.00_11.18_	95.00_11.18_	**100.00_0.00_**	**100.00_0.00_**
Breast-Tissue	60.00_27.89_	40.00_36.51_	46.67_29.81_	60.00_27.89_	53.33_29.81_	60.00_27.89_	53.33_29.81_	60.00_27.89_	**73.36_13.32_**
UKM	88.00_13.04_	92.00_13.04_	90.00_10.00_	94.00_8.94_	88.00_16.43_	86.00_13.42_	86.00_13.42_	94.00_8.94_	**96.00_5.76_**
Vertebral-Column	65.00_16.03_	65.00_19.00_	60.00_19.90_	61.67_18.26_	66.67_5.89_	68.33_22.36_	66.67_28.87_	86.67_9.50_	**90.67_6.02_**
Ecoli	65.00_37.91_	70.00_27.39_	55.00_32.60_	70.00_27.39_	55.00_32.60_	75.00_30.62_	90.00_13.69_	**90.00_13.69_**	**90.00_12.40_**
Average	71.00	71.01	70.53	72.65	72.09	71.67	75.67	81.95	**85.23**
Rank Avg.	6.33	5.89	6.39	5.11	5.78	5.89	5.28	2.56	**1.78**

The best result is in bold face.

**Table 3:** Comparison of $P_{avg}$ (%) for every oversampling scheme on different data sets.

Data Set	Baseline	SSMOTE	GCS	ABNC	OSMOTE	MDO	MDO+	AMDO	EMDO
Balance	56.38_1.75_	58.38_2.94_	56.65_4.03_	62.33_3.29_	57.86_2.78_	57.28_2.92_	55.45_1.66_	60.37_2.06_	**64.38_3.59_**
Hayes-Roth	84.91_6.71_	84.67_5.06_	85.33_5.58_	83.52_7.08_	84.97_6.74_	84.91_6.71_	84.97_6.74_	84.97_6.74_	**85.61_2.39_**
New-Thyroid	88.86_6.02_	91.08_3.02_	93.14_4.55_	93.59_1.45_	92.54_6.61_	89.81_6.84_	94.98_3.59_	96.54_2.99_	**96.60_2.05_**
Page-Blocks	84.30_2.14_	84.57_1.88_	88.77_4.49_	79.70_5.57_	89.61_2.98_	81.24_1.79_	86.13_2.48_	88.77_1.92_	**90.15_1.87_**
Dermatology	95.67_2.05_	95.73_1.83_	93.50_2.71_	97.10_0.75_	95.31_2.45_	95.67_2.05_	96.06_1.62_	96.88_0.26_	**97.13_0.20_**
Breast-Tissue	63.22_3.74_	60.89_3.94_	68.78_5.77_	66.00_4.88_	65.83_7.05_	63.22_3.74_	66.56_2.17_	63.22_3.74_	**70.60_6.28_**
UKM	92.18_2.02_	92.57_4.85_	91.03_2.00_	94.49_2.45_	91.78_2.32_	91.45_2.48_	91.92_2.50_	94.23_2.14_	**95.24_1.37_**
Vertebral-Column	76.44_2.65_	77.22_4.66_	75.67_5.86_	76.67_3.12_	77.00_4.71_	78.22_5.55_	76.56_7.41_	81.89_2.38_	**85.77_1.53_**
Ecoli	74.64_7.88_	72.81_12.01_	72.73_11.35_	76.23_7.14_	73.12_8.72_	77.24_7.03_	82.30_5.21_	82.44_5.08_	**85.31_1.62_**
Average	79.61	79.77	80.62	81.07	80.89	79.89	81.66	83.26	**85.65**
Rank Avg.	7.00	6.11	6.17	4.78	5.44	6.33	4.89	3.28	**1.00**

The best result is in bold face.

**Table 4:** Comparison of AUCm (%) for every oversampling scheme on different data sets.

Data Set	Base	SSMOTE	GCS	ABNC	OSMOTE	MDO	MDO+	AMDO	EMDO
Balance	56.95_1.91_	58.12_3.27_	57.10_3.43_	60.52_2.89_	58.58_3.18_	57.40_2.78_	56.60_0.88_	60.61_1.24_	**65.61_4.30_**
Hayes-Roth	94.34_2.52_	94.25_1.90_	94.50_2.09_	93.82_2.65_	94.36_2.53_	94.34_2.52_	94.36_2.53_	94.36_2.53_	**94.67_2.81_**
New-Thyroid	91.40_5.33_	93.90_4.45_	95.43_3.76_	95.76_2.37_	94.04_5.11_	91.85_6.74_	97.04_2.94_	98.37_1.41_	**98.55_1.23_**
Page-Blocks	90.75_3.57_	89.84_2.16_	94.81_4.66_	86.82_7.87_	94.96_3.16_	87.90_4.66_	93.97_2.91_	95.51_2.07_	**96.07_2.24_**
Dermatology	97.32_3.46_	97.39_3.27_	95.55_4.17_	99.05_0.28_	96.09_4.09_	97.32_3.46_	97.48_3.23_	98.98_0.26_	**99.06_0.22_**
Breast-Tissue	76.80_7.31_	72.18_10.10_	75.80_9.99_	78.30_8.92_	76.92_9.75_	76.80_7.31_	77.30_8.09_	76.80_7.31_	**82.37_3.77_**
UKM	94.14_3.65_	94.94_5.27_	94.19_3.04_	96.41_2.61_	94.01_4.64_	93.333.88	93.55_3.92_	96.45_3.02_	**97.26_2.49_**
Vertebral-Column	79.25_3.17_	79.96_5.56_	78.38_6.36_	79.42_5.04_	80.04_2.48_	81.13_6.76_	79.54_8.76_	86.04_1.72_	**89.07_2.00_**
Ecoli	83.07_11.54_	83.43_10.88_	79.78_11.44_	84.78_9.21_	79.74_11.00_	86.38_9.56_	91.97_4.61_	91.59_4.19_	**92.91_2.92_**
Average	84.89	84.89	85.06	86.10	85.42	85.16	86.87	88.75	**90.62**
Rank Avg.	7.00	6.22	6.33	4.89	5.44	6.33	4.89	2.89	**1.00**

The best result is in bold face.

**Table 5:** Comparison of MAUC (%) for every over sampling scheme on different data sets.

Data Set	Baseline	SSMOTE	GCS	ABNC	OSMOTE	MDO	MDO+	AMDO	EMDO
Balance	67.29_1.31_	68.79_2.20_	67.49_3.02_	71.75_2.47_	68.39_2.09_	67.96_2.19_	66.59_1.25_	70.27_1.54_	**73.13_2.77_**
Hayes-Roth	88.68_5.03_	88.50_3.79_	89.00_4.18_	87.64_5.31_	88.73_5.06_	88.68_5.03_	88.73_5.06_	88.73_5.06_	**89.15_4.86_**
New-Thyroid	91.64_4.52_	93.31_2.27_	94.86_3.41_	95.19_1.09_	94.40_4.96_	92.36_5.13_	96.24_2.69_	97.40_2.24_	**97.76_2.14_**
Page-Blocks	90.19_1.34_	90.35_1.18_	92.98_2.80_	87.31_3.48_	93.51_1.86_	88.27_1.12_	91.33_1.55_	92.98_1.20_	**93.85_1.17_**
Dermatology	97.40_1.23_	97.44_1.10_	96.10_1.62_	**98.26_0.45_**	97.19_1.47_	97.40_1.23_	97.63_0.97_	98.13_0.16_	**98.26_0.16_**
Breast-Tissue	77.93_2.24_	76.53_2.36_	81.27_3.46_	79.60_2.93_	79.50_4.23_	77.93_2.24_	79.93_1.30_	77.93_2.24_	**84.07_5.16_**
UKM	94.79_1.34_	95.05_3.23_	94.02_1.33_	96.32_1.64_	94.52_1.54_	94.30_1.65_	94.61_1.67_	96.15_1.42_	**96.91_1.05_**
Vertebral-Column	82.33_1.99_	82.92_3.50_	81.75_4.39_	82.50_2.34_	82.75_3.53_	83.67_4.16_	82.42_5.55_	86.42_1.78_	**89.05_1.33_**
Ecoli	84.15_4.93_	83.01_7.50_	82.96_7.10_	85.15_4.46_	83.20_5.45_	85.77_4.39_	88.93_3.26_	89.03_3.18_	**90.82_1.01_**
Average	86.04	86.21	86.71	87.08	86.91	86.26	87.38	88.56	**90.33**
Rank Avg.	7.00	6.11	6.17	4.72	5.44	6.33	4.89	3.28	**1.06**

The best result is in bold face.

**Table 6:** Characteristics of the data sets for simulations in Example 2.

Data Set	Size	Attributes	Classes	Class Distribution	IR_max_	No. of Ellipsoids
Statlog (Shuttle)	58000	9	7	45586/50/171/8903/ 3267/10/13	5684.67	NA/3/4/5/5/ 3/1
Mafalda	23762	14	3	17757/2990/3015	5.94	NA/11/11

NA: not available.

**Table 7:** Comparison of the performance with and without EMDO for imbalanced sensory data.

Data Set		*P_min_*	*P_avg_*	AUCm	MAUC
Statlog (Shuttle)	w/ EMDO	89.33_13.73_	96.60_1.95_	99.66_0.47_	99.32_0.94_
	w/o EMDO	60_48.99_	93.21_7.42_	77.65_6.17_	92.80_1.66_
Mafalda	w/ EMDO	57.38_18.33_	72.34_9.33_	76.39_9.31_	78.19_8.22_
	w/o EMDO	33.17_12.13_	60.77_10.53_	65.25_7.88_	67.80_6.99_
